# Dolichoectasia of the ophthalmic artery: a case report on the treatment strategy in endovascular therapy and literature review

**DOI:** 10.1186/s12872-024-03771-9

**Published:** 2024-02-10

**Authors:** Tomoya Oishi, Hiroaki Neki, Tomoya Sakamoto, Muneaki Hashimoto, Yuichi Mochizuki, Yoshinobu Kamio, Kazuhiko Kurozumi

**Affiliations:** Department of Neurosurgery, Hamamatsu University School of Medicine, 1-20-1 Handayama, Chuo-Ku, Hamamatsu City, Shizuoka, 431-3192 Japan

**Keywords:** Dolichoectasia, Ophthalmalgia, Ophthalmic artery, Endovascular therapy

## Abstract

**Background:**

Dolichoectasia is a rare arterial condition characterized by the dilatation, tortuosity, and elongation of cerebral blood vessels. The vertebrobasilar artery and internal carotid artery are the common sites of dolichoectasia. However, dolichoectasia of the branch arteries, such as the ophthalmic artery (OA), is extremely rare. To the best of our knowledge, this is the first case of ophthalmic dolichoectasia that was successfully treated with endovascular internal coil trapping.

**Case presentation:**

A 54-year-old female patient presented with transient left ophthalmalgia and visual disturbance. Magnetic resonance imaging revealed a dilated and elongated left OA compressing the optic nerve at the entrance of the optic canal. However, a previous image that was taken 17 years back revealed that the OA was normal, which suggested the change in dolichoectasia was acquired. Cerebral angiography showed that the dilated and tortuous OA was running from the ophthalmic segment of the left internal carotid artery into the orbit. The symptoms could have been attributed to the direct compression of the dolichoectatic OA in the optic canal. A sufficient anastomosis between the central retinal artery and the middle meningeal artery was identified on external carotid angiography with balloon occlusion of the internal carotid artery. Endovascular treatment with internal trapping of the OA was performed due to ophthalmic symptom progression. Internal coil trapping of the OA was performed at the short segment between the OA bifurcation and the entrance of the optic canal. As expected, the central retinal artery was supplied via the middle meningeal artery after the treatment. The transient visual disturbance was immediately resolved. Ophthalmalgia worsened temporarily after the treatment. However, it completely resolved after several days of oral corticosteroid therapy. Postoperative angiography showed that the origin of the OA was occluded and that the OA in the optic canal was shrunk. The flow of the central retinal arteries via the middle meningeal artery was preserved.

**Conclusions:**

OA dolichoectasia is rare, and its pathogenesis and long-term visual prognosis are still unknown. However, endovascular therapy can improve symptom by releasing the pressure site in the optic canal.

## Background

Intracranial arterial dolichoectasia is a rare condition characterized by the dilatation, elongation, and tortuosity of cerebral blood vessels. One of its causes is the degeneration of the internal elastic lamina caused by arteriosclerosis or other unexplained conditions [1, 2]. The incidence rate of dolichoectasia is 1.3%–4.4%, and the relevant factors of dolichoectasia are atherosclerosis, hypertension, age, smoking, and connective tissue diseases [3–5]. Dolichoectasia is more likely to occur in large vessels such as the vertebrobasilar artery and internal carotid artery (ICA) and is less commonly observed in branch arteries [6, 7]. Although the condition is frequently asymptomatic, it can become symptomatic, with ischemic, and hemorrhagic events caused by thrombosis or vessel wall thinning. Moreover, dolichoectasia of the large vessels occasionally presents with compression symptoms of the cranial nerves, such as the trigeminal nerve, facial nerve, and optic nerves [2, 8, 9]. Dolichoectasia treatment with cranial nerve compression involves surgical procedures to relieve nerve compression and/or reduce mass effect. Dolichoectasia of the ophthalmic artery (OA) is extremely rare. Thus, a consensus on treatment indication and methods has not been established. Herein, we present a case of dolichoectasia of the OA and perform a literature review.

## Case presentation

**Fig. 1:**
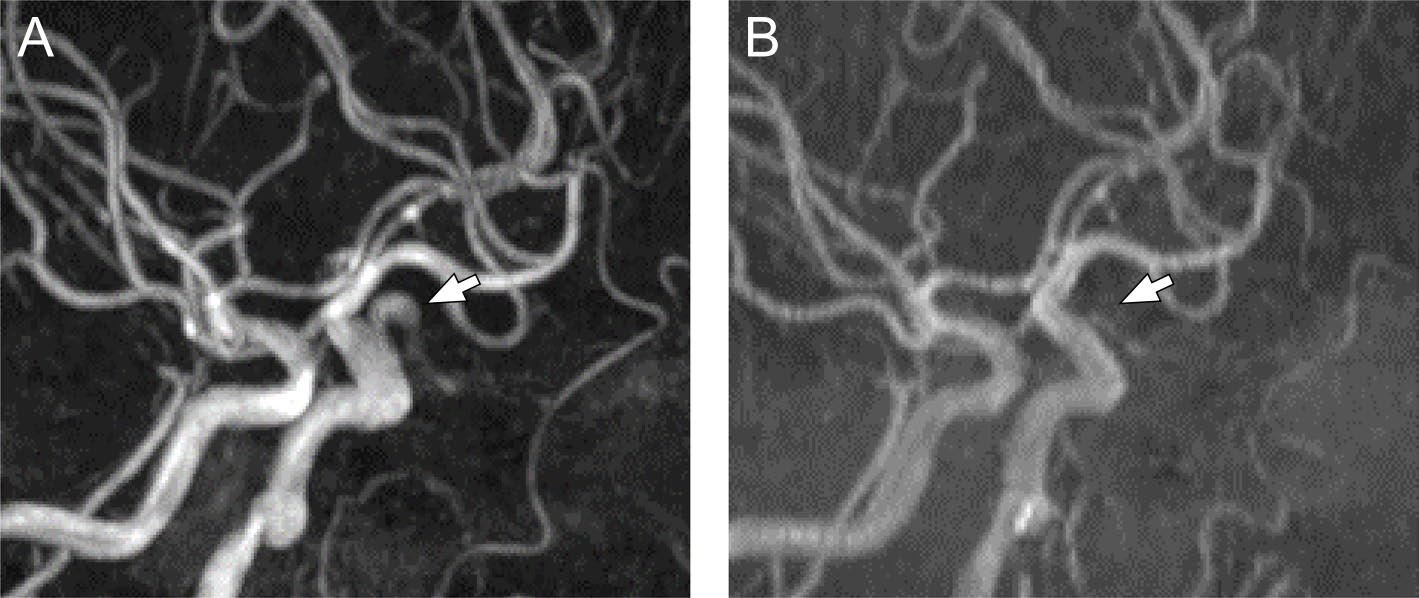
Preoperative magnetic resonance angiography (MRA). MRA showing a dilated and tortuous left ophthalmic artery (OA) (**A**, arrow). MRA performed 17 years back revealed no abnormality in the left OA (**B**, arrow)

**Fig. 2:**
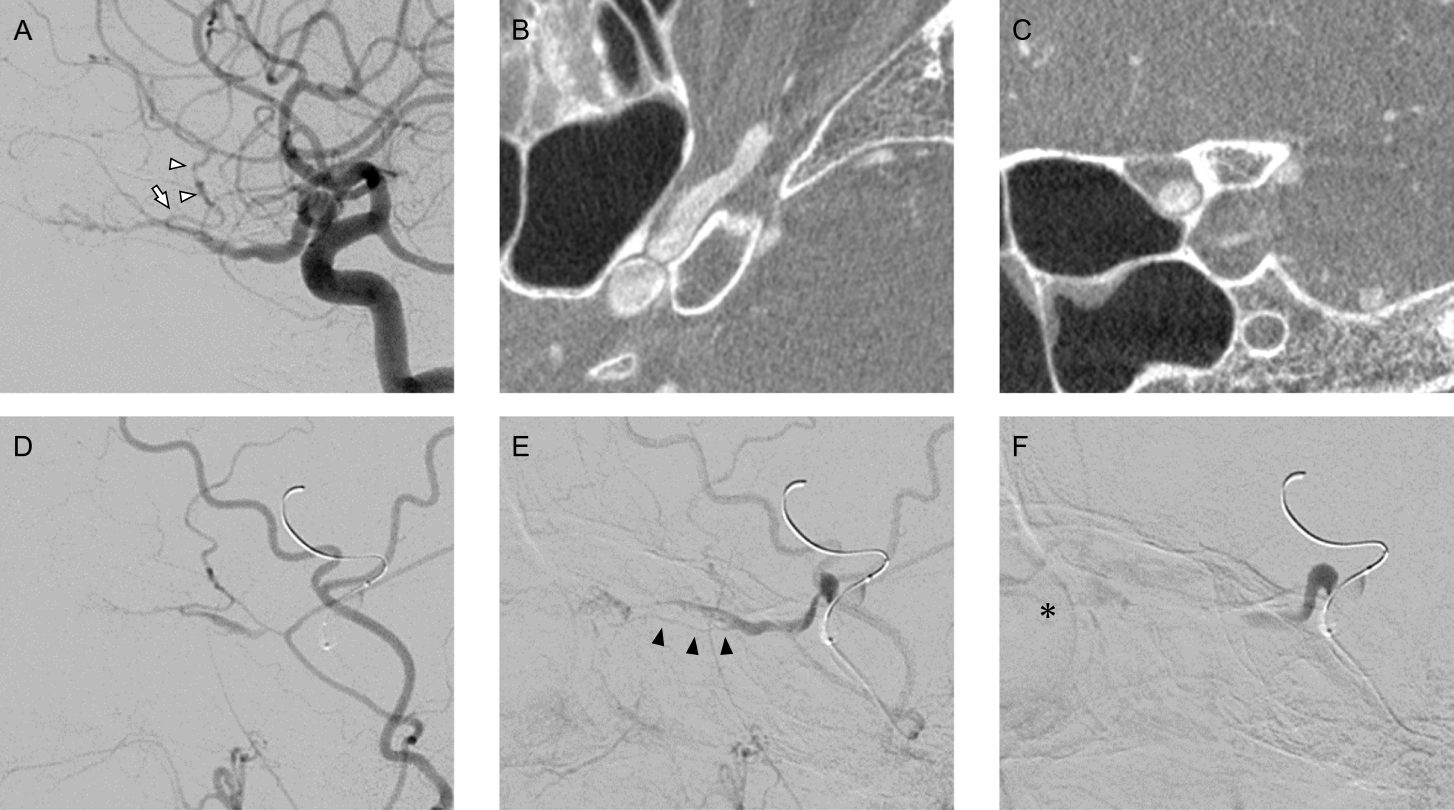
Images of preoperative cerebral angiography of the dolichoectatic OA and the test of balloon occlusion. Preoperative cerebral angiography showed the dolichoectatic left ophthalmic artery (OA). Lateral projection of the left internal carotid arteriography (ICAG) revealed that the frontal branch of the middle meningeal artery (MMA) (arrowhead) was supplied from the dilated OA via the anastomotic artery (arrow) between OA and MMA (**A**). Maximum intensity projection (MIP) images obtained from the left ICAG showed that the OA was tortuous and dilated in the optic canal (**B**: axial, **C**: coronal). Left ECAG with balloon occlusion of the left ICA at the C3 segment revealed retrograde visualization of the proximal segment of the OA and central retinal arteries via the MMA (**D**: early phase, **E**: late phase) and central retinal arteries (black arrowhead). A retinal crescent (asterisk) was observed at the late venous phase (**F**)

A 54-year-old female patient presented to our department due to recurrent transient left ophthalmalgia accompanied by visual disturbance. The patient had no significant medical or family history. Ophthalmologic examination revealed neither evident abnormal retinal findings nor visual field defects. However, flicker in the left eye decreased to 30.3 Hz and that of the right eye to 40.2 Hz, thereby indicating a slight impairment in the left optic nerve. Magnetic resonance angiography (MRA) revealed that the left OA was dilated and elongated (Fig. 1A). However, 17 years ago, an image obtained at the time of examination for bifrontal headache revealed that the OA was normal (Fig. 1B). Similar to MRA, left internal carotid arteriography (ICAG) showed that the left OA was tortuous upward just proximal to the optic canal (Fig. 2A) and was dilated to a maximum diameter of 3.90 mm. The maximum intensity projection (MIP) images revealed that the dolichoectatic OA was running through the optic canal from the entry (Fig. 2B and C). The optic canal was filled with the dilated OA, and the optic nerve was compressed. There was no evident venous drainage indicating arteriovenous malformation or dural arteriovenous fistula. Left external carotid angiography (ECAG) with balloon occlusion of the ipsilateral ICA at the orbital bifurcation showed retrograde visualization of the proximal segment of the OA and central retinal arteries via the middle meningeal artery (MMA). Moreover, a retinal crescent was observed (Fig. 2D-F).

**Fig. 3:**
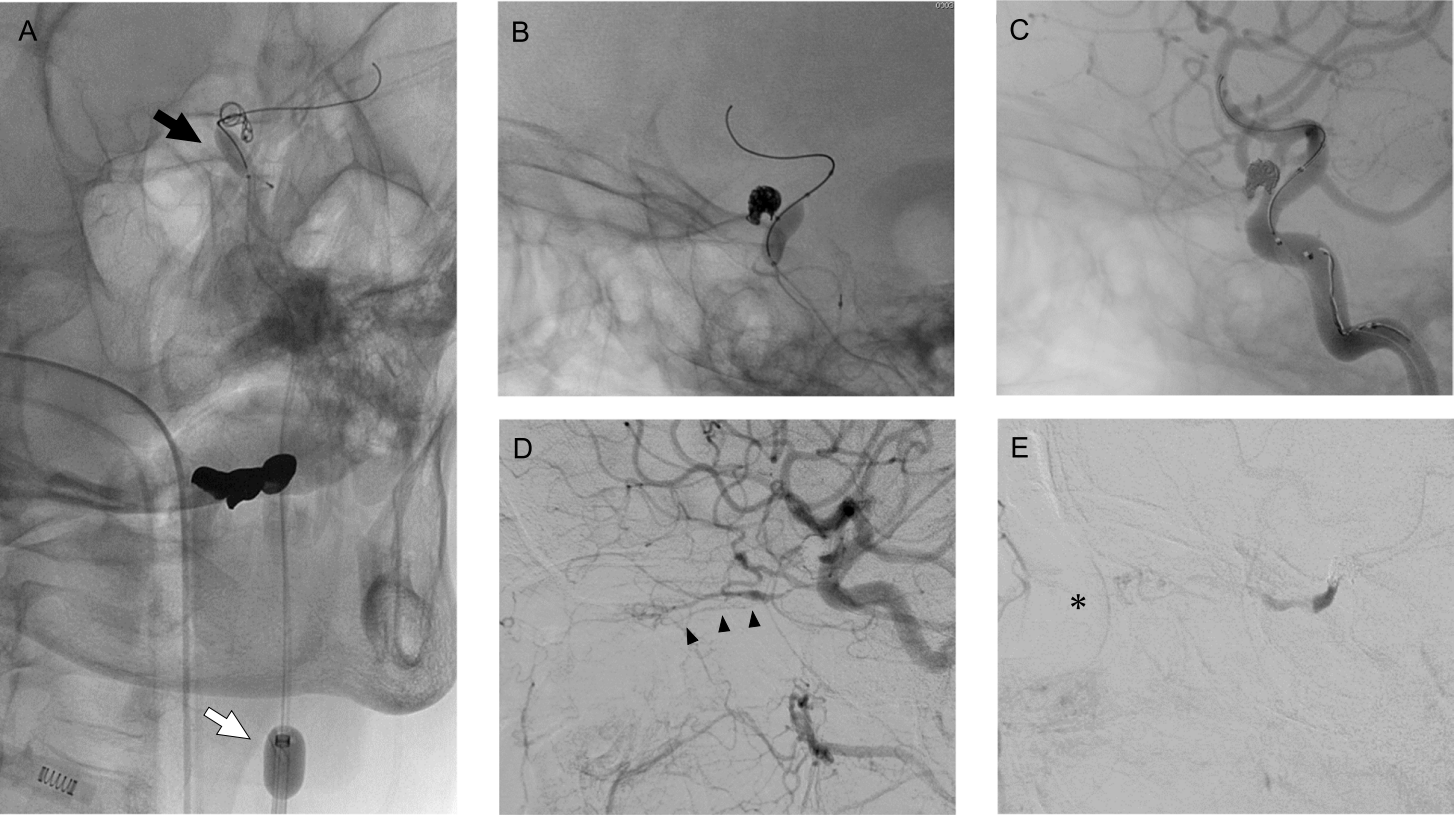
Images of intraoperative cerebral angiography. Right oblique projection of the radiographic image showed double-balloon flow restoration with a balloon-guided catheter placed at the internal carotid artery (ICA) (white arrow) and balloon catheter at the ICA-ophthalmic artery (OA) bifurcation (black arrow). Moreover, the framing coil was placed from the microcatheter in the OA (**A**). Lateral projection of the radiographic image (**B**) and left internal carotid arteriography (**C**) revealed that the compact coil mass was finally placed at the short segment at the origin of the OA. Lateral projection of the common carotid arteriography showed no prograde flow from the ICA into the OA, and the proximal segment of the OA and the central retinal artery (black arrowhead) (**D**) were retrogradely supplied from the recurrent meningeal artery from the middle meningeal artery. Further, the retinal crescent (asterisk) was detected (**E**)

Ophthalmalgia and visual disturbance exacerbated; thus, endovascular treatment was carried out. Dual antiplatelet agents (aspirin and clopidogrel) were administered for 2 weeks as preoperative medications. Under general anesthesia, an 8-Fr balloon-guided catheter was advanced into the left ICA via the right groin puncture. An occlusion balloon catheter was placed at the ICA-OA bifurcation, and the microcatheter was navigated into the OA (Fig. 3A). Via dual balloon occlusion with a balloon-guided catheter and balloon catheter, the OA was embolized by the coils in a short segment from the origin to the entrance of the optic canal (Fig. 3B and C). Postembolization common carotid arteriography showed complete occlusion of the OA. Meanwhile, the proximal segment of the OA was retrogradely supplied from the MMA, and the central retinal arteries and retinal crescent were observed (Fig. 3D and E).

**Fig. 4:**
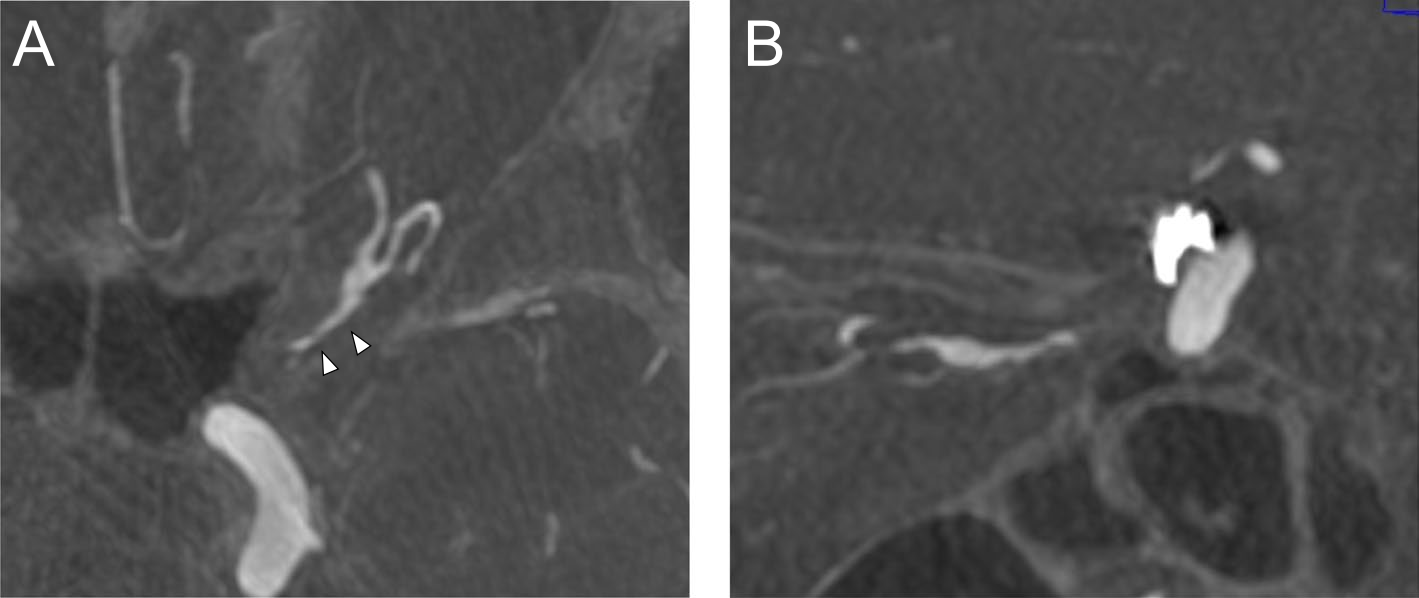
Images of postoperative cerebral angiography 6 month after the endovascular treatment. Maximum intensity projection images obtained 6 months after embolization showed improvement in dilation of the ophthalmic artery in the optic canal (**A**: axial, **B**: sagittal). The coil mass did not change after the treatment

There was no visual impairment during the postoperative course, and the patient had improvement in transient ophthalmalgia a few days after oral steroid therapy. Ophthalmalgia and visual disturbance were completely resolved. Angiography was performed 6 months after endovascular embolization. Results showed that the origin of the OA was occluded and that the distal flow of the OA via the MMA was preserved. On the MIP image, the proximal segment of the OA in the optic canal became smaller compared with that before embolization (Fig. 4A and B).

## Discussion and conclusions

Dolichoectasia of the OA is an extremely rare condition. Notably, endovascular embolization of the OA itself can be achieved without visual acuity worsening via the preoperative evaluation of blood supply to the central retinal artery from the anastomosis to the MMA.

There are different criteria for classifying posterior dolichoectasia [10, 11]. For the anterior circulation, the diagnostic criteria for dolichoectasia are not well defined. Passero et al. defined ectasia as an ICA of ≥ 7 mm, MCA of ≥ 4 mm, and VA of ≥ 4 mm [1]. However, there are no clear criteria for the definition of dilatation based on the diameter of the peripheral arteries such as the OA. Zhai et al. have defined dolichoectasia as a vessel diameter of ≥ 2 SD [5]. The mean diameter of the OA was 1.38 (± 0.23) mm, with a median of 1.35 mm [12]. Therefore, the OA in the current case was dilated to 3.90 mm, thereby sufficiently indicating dolichoectasia.

The clinical manifestations of optic neuropathy caused by dolichoectasia of the ICA are optic disk edema and optic nerve pallor. The duration of vision loss before diagnosis ranged from 3 weeks to 21 years [13]. In this case, the time from onset to diagnosis was approximately 2 months, and there were no findings of optic neuropathy in the fundus. Paraclinoid aneurysms are often characterized by visual symptoms caused by optic nerve compression. However, the patient presented with intermittent visual field disturbance, which might have been an early symptom of optic neuropathy attributed to vascular compression attributed to dolichoectatic OA. The symptom of phosphene (flashes of light) is an early symptom of optic nerve compression by the anterior communicating artery aneurysm [14].

The pathogenesis of symptoms is important in determining the therapeutic target and strategy. In general, the mechanisms associated with visual disturbance caused by lesions in the paraclinoid area are as follows: 1) direct optic nerve compression attributed to the ecstatic vessel, 2) optic nerve irritation and dysfunction due to vessel pulsatility, and 3) indirect compression from above caused by the falciform ligament similar to the knife edge [15, 16]. In this case, the optic canal was occupied by a prominently dilated OA in the MIP image of the left ICAG. Hence, the symptoms could have been attributed to the direct compression of the dolichoectatic OA in the optic canal. In relation to this, symptomatic improvement was observed with coil embolization. However, the positional association between the coil mass in the OA and the optic nerve did not change. Postoperative ophthalmalgia may have been exacerbated by OA thrombosis. This is similar to the cranial nerve symptom worsening caused by thrombosis after the endovascular treatment of aneurysms with flow diverters [17]. The symptoms finally improved because the OA in the optic canal was not embolized.

In this case, two strategies were used to release optic nerve compression at the optic canal entrance. First, surgical unroofing of the optic canal was utilized to release optic nerve compression including that at the entrance [16]. Second, endovascular embolization in the OA with a short segment was applied to the optic canal entrance to facilitate distal vessel shrinkage. The second strategy was feasible due to the presence of anastomosis from the ECA via the dilated MMA. Preoperative ECAG with ICAG balloon occlusion revealed that the OA could be safely embolized without visual complication by evaluating the collateral blood supply from the ECA to the OA and central retinal artery. Visual complications can be prevented despite OA occlusion if the collaterals of the ECA are sufficient under balloon occlusion of the OA [18]. However, it is challenging to occlude the OA without causing visual deterioration if the collateral vessels are not well developed. In the current case, as expected, sufficient blood flow from the MMA to the OA, as observed in balloon occlusion, resulted in adequate retrograde blood flow to the OA after embolization.

The incidence rate of visual complications during coil embolization of IC-ophthalmic aneurysms is 10.4%, and the complications are believed to be caused by microemboli into the central retinal artery during coil embolization [19, 20]. In this case, a balloon-guided catheter as well as a balloon catheter was used with strict heparinization to obtain complete IC occlusion, because complete inhibition of prograde blood flow in the OA is necessary for preventing thrombus migration into retinal artery during embolization.

We performed a literature search on PubMed to identify other cases of dolichoectasia of the OA. We only found two reports on dolichoectasia of the OA. In the two cases, the patients were treated with steroids. However, in the current case, the patient underwent endovascular coiling (Table 1). Khanna et al. showed a case of compression optic neuropathy associated with dolichoectatic OA treated with steroids. Methotrexate temporarily improved vision, resulting in partial visual defect [21]. However, in one case of intraorbital dolichoectatic OA, visual acuity worsened despite steroid treatment [22]. Thus, to the best of our knowledge, this is the first case of ophthalmic dolichoectasia that was successfully treated with endovascular internal coil trapping. Although the number of cases was limited and the long-term prognosis of dolichoectasia of the OA is still unknown, endovascular embolization could be effective against this disease. Cautious follow-up is required because visual complications in the distance phase have been reported with coil embolization.

**Table 1:** Summary of three cases of dolichoectasia of the ophthalmic artery

Author, year	Age, sex	Symptoms	Treatment	Visual outcomes
Khanna RK et al., 2019 [21]	52, F	Proptosis	Steroid, methotrexate	Partial loss
Gire J et al., 2010 [22]	47, F	Progressive visual loss	Steroid	Moderate decrease in visual acuity
Current case, 2023	54, F	Ophthalmalgia, visual loss attacks	endovascular	Good

The table shows the summary of dolichoectasia of the OA. All patients are middle-aged females. In the two cases, the patients were treated with steroids. However, in the current case, endovascular trapping achieved a favorable outcome

Ophthalmic dolichoectasia is an extremely rare condition, and the pathogenesis, and long-term prognosis of dolichoectasia of the OA are still unknown. In this case, the collateral blood flow to the central retinal artery was well developed. Therefore, the symptoms improved by coil trapping the dolichoectatic OA proximal to the optic canal wherein the optic nerve was compressed.

## Data Availability

The datasets used during the current study are available from the corresponding author on reasonable request.

## Abbreviations

OA: Ophthalmic artery
ICA: Internal carotid artery
ECA: External carotid artery
MMA: Middle meningeal artery
MRA: Magnetic resonance angiography
ICAG: Internal carotid arteriography
ECAG: External carotid arteriography
MIP: Maximum intensity projection
